# The Peach RGF/GLV Signaling Peptide pCTG134 Is Involved in a Regulatory Circuit That Sustains Auxin and Ethylene Actions

**DOI:** 10.3389/fpls.2017.01711

**Published:** 2017-10-11

**Authors:** Nicola Busatto, Umberto Salvagnin, Francesca Resentini, Silvia Quaresimin, Lorella Navazio, Oriano Marin, Maria Pellegrini, Fabrizio Costa, Dale F. Mierke, Livio Trainotti

**Affiliations:** ^1^Department of Biology, University of Padova, Padova, Italy; ^2^Department of Genomics and Crop Biology, Research and Innovation Centre, Fondazione Edmund Mach, Trento, Italy; ^3^Department of Biomedical Sciences, University of Padova, Padova, Italy; ^4^Department of Chemistry, Dartmouth College, Hanover, NH, United States

**Keywords:** *Arabidopsis thaliana*, CLE-LIKE (CLEL), fruit ripening, GOLVEN (GLV), *Nicotiana tabacum*, peptide hormone, *Prunus persica*, ROOT GROWTH FACTOR (RGF)

## Abstract

In vascular plants the cell-to-cell interactions coordinating morphogenetic and physiological processes are mediated, among others, by the action of hormones, among which also short mobile peptides were recognized to have roles as signals. Such peptide hormones (PHs) are involved in defense responses, shoot and root growth, meristem homeostasis, organ abscission, nutrient signaling, hormone crosstalk and other developmental processes and act as both short and long distant ligands. In this work, the function of *CTG134*, a peach gene encoding a ROOT GROWTH FACTOR/GOLVEN-like PH expressed in mesocarp at the onset of ripening, was investigated for its role in mediating an auxin-ethylene crosstalk. In peach fruit, where an auxin-ethylene crosstalk mechanism is necessary to support climacteric ethylene synthesis, *CTG134* expression peaked before that of *ACS1* and was induced by auxin and 1-methylcyclopropene (1-MCP) treatments, whereas it was minimally affected by ethylene. In addition, the promoter of *CTG134* fused with the GUS reporter highlighted activity in plant parts in which the auxin-ethylene interplay is known to occur. *Arabidopsis* and tobacco plants overexpressing CTG134 showed abnormal root hair growth, similar to wild-type plants treated with a synthetic form of the sulfated peptide. Moreover, in tobacco, lateral root emergence and capsule size were also affected. In *Arabidopsis* overexpressing lines, molecular surveys demonstrated an impaired hormonal crosstalk, resulting in a re-modulated expression of a set of genes involved in both ethylene and auxin synthesis, transport and perception. These data support the role of pCTG134 as a mediator in an auxin-ethylene regulatory circuit and open the possibility to exploit this class of ligands for the rational design of new and environmental friendly agrochemicals able to cope with a rapidly changing environment.

## Introduction

Plants are complex multicellular organisms requiring the coordination of a wide range of processes related to growth, reproduction and stress responses. For decades hormones, such as auxin, ethylene and abscisic acid have been considered as the primary chemical signals involved in the intracellular communications in higher plants. The action of these hormones depends not only on the cellular context, but also on the relationship established among them. To date, the hormonal crosstalk has been mainly investigated in *Arabidopsis*, which shed light, among others, on the crosstalk between auxin and ethylene (Van de Poel et al., 2015). The first, most evident and studied effect of their interaction is about the regulation of root morphogenesis. Indeed, in this organ it has been demonstrated that root hair formation, elongation (Pitts et al., 1998; Dolan, 2001) and differentiation, together with the development of lateral roots are regulated by the interplay occurring between auxin and ethylene (Zhang et al., 2016).

In addition to the traditional hormones, a plethora of secreted and non-secreted peptides have been recognized as regulators of various aspects of plant growth, including defense responses, callus growth, meristem organization, self-incompatibility and fertilization, organ abscission and shoot and root development [reviewed in (Matsubayashi, 2014; Tavormina et al., 2015)]. Moreover peptide hormones (PHs) can synergically interact with hormones establishing functional interplay. Cellular and genetic evidences have shown a physiological connection between hormones and PHs. For instance, ROOT GROWTH FACTOR/GOLVEN/CLE-Like (RGF/GLV/CLEL) peptides can alter auxin gradients by changing the turnover of IAA carriers (Whitford et al., 2012). In *Arabidopsis* the RGF/GLV/CLEL family codes for secretory peptides and includes 11 genes with similar structure (Matsuzaki et al., 2010; Whitford et al., 2012) and undergoing specific post-translation modifications by sulfation of a conserved Tyr residue and hydroxylation of a Pro residue. In *Arabidopsis*, RGF/GLV/CLEL mutants shown an impaired gravitropic response in root hypocotyl.

Despite the importance of this regulatory mechanism, the biology of PHs is still in its infancy, especially in non-model but agronomically relevant species. A putative PH in particular, namely CTG134 GLV-like, was identified in peach through a comprehensive transcriptomic survey (Tadiello et al., 2016). This gene resulted to be expressed at the transition between preclimacteric and climacteric stage in peach fruit. Moreover, while CTG134 was induced by exogenous treatment of 1-methylcyclopropene (1-MCP), an ethylene competitor largely used to delay the normal physiological ripening progression (Watkins, 2006), its expression was also totally repressed in ripe fruit of *stony hard*, a peach mutant showing impairment both in ethylene production and cell wall disassembly metabolism (Pan et al., 2015). *Prunus persica* is a fleshy climacteric fruit, whose ripening syndrome extensively relies on the presence of a burst in the production of the plant hormone ethylene accompanied by a respiratory increase occurring at the late stage of fruit ripening (Looney et al., 1974). The molecular mechanism underlying the transition of ethylene synthesis from auto-inhibitory system 1 (used for basal level of hormone synthesis) to autocatalytic system 2 (used for large production of the hormone) is still far from being fully understood, but the need of auxin is strongly supported (Miller et al., 1987; Trainotti et al., 2007; Tatsuki et al., 2013; Pan et al., 2015; Tadiello et al., 2016). In this work, the functional validation of pCTG134 was carried out in *Arabidopsis* and tobacco, providing new evidence about its role as a major regulator in the auxin/ethylene crosstalk. This and other functions of RGF/GLV/CLEL peptides suggest the possibility for the rational design of novel and environmental friendly agrochemicals with interesting potentials to control fruit quality and post-harvest life in a rapidly changing environment.

## Materials and Methods

### Plant Materials

Peach fruits were collected from cv. ‘Redhaven’ (RH) and cv. “Stark Red Gold” (SRG) as described in Tadiello et al. (2016). RH peaches at different stages of development [i.e., S1, S2, S3I, S3II, S4I, and S4II, corresponding to 40, 65, 85, 95, 115, and 120 days after full bloom (dAFB), respectively] were collected early in the morning and mesocarps were frozen in liquid nitrogen after removal of the peel and stored at -80°C until used. Fruits for qRT-PCR analyses were the same used in Tadiello et al. (2016), while those used for the *in situ* hybridization were collected in 2015. The heterologous CTG134 overexpression was carried out in *Arabidopsis* and tobacco plants. Seeds of *Arabidopsis thaliana* Columbia accession (Col-0) were surface-sterilized, stratified overnight at 4°C and germinated on plant growth medium (Murashige and Skoog, 1962) or in potting soil at 22°C. To characterize root growth, MS plates were tilted with an angle of 45°. *Nicotiana tabacum* SNN plants were instead grown following standard protocols in controlled greenhouse.

### Hormone Treatments on Redhaven Fruit

Hormone treatments were performed at 22 °C on Redhaven (RH) fruits at S3II, corresponding to 95 days after full bloom (dAFB), attached to a branch that was kept in water. The auxin treatment was performed by dipping the whole fruit in 1-naphthalene acetic acid [NAA, 2 mmol L^-1^ added with Silwet L-77 (200 μL L^-1^) as surfactant] for 15 min; thereafter, fruit were sprayed with the NAA solution every 12 h over a period of 48 h (NAA omitted in the mock control). The ethylene treatment was instead carried out by placing whole fruit in a sealed chamber and flushing them with ethylene (10 μL L^-1^) in air at a flow rate of approximately 6 L h^-1^ as previously described in Tadiello et al. (2016). At the end of treatment (48 h), mesocarp tissues from a pool of 12 fruit per class were frozen in liquid nitrogen and stored at -80°C until used.

### 1-MCP Treatments on Stark Red Gold Fruit

Stark Red Gold (SRG) peaches were harvested at 123 dAFB (S4), i.e., at commercial maturity date, which is about 2 weeks later than that of RH. In order to obtain homogeneous fruit at different stages of ripening, fruits were graded immediately after harvest into three classes by decreasing ranges of the index of absorbance difference (I_AD_; class 0: I_AD_ 1.2–0.9; class 1: I_AD_ 0.9–0.6; class 2: I_AD_ 0.6–0.3), as previously described (Ziosi et al., 2008). According to previous studies (Ziosi et al., 2008), fruit from the three classes could be classified as belonging to pre-climacteric (class 0), onset of climacteric (class 1), and full climacteric (class 0) stages of the ripening process. Fruits from each class were treated or not (controls) with 1-methylcyclopropene (1-MCP). Treatment was carried by placing one hundred fruits per class in two sealed 30-L plastic jars (50 fruit each). SmartFresh^TM^ (AgroFresh Inc., Philadelphia, PA, United States), a commercial powder containing 0.14% (w/w) 1-MCP a.i., was prepared as a 10-fold concentrated stock solution following the technical bulletin of the company, and injected as 10 mL of air (final concentration 1 mL L^-1^ equivalent to 1 μL L^-1^). The same total number of fruit per class was kept in two sealed jars for 12 h at 25°C without 1-MCP (air controls). Treated and control fruit were then transferred to a growth chamber at 25°C. At the end of treatment (12 h) and at each following sampling time, mesocarp tissues from a pool of 10 fruit per class were frozen in liquid nitrogen and stored at -80°C until used, as described in Tadiello et al. (2016).

### RNA Extraction and Expression Analyses by Quantitative Real Time PCR (qRT-PCR)

Peach RNA was prepared from a frozen powder obtained by grinding mesocarp sectors from at least four different fruits. From 4 g of this powder, total RNA was extracted following a protocol previously described (Chang et al., 1993). *Arabidopsis* RNA was extracted from wild type and 35S::CTG134 mutant seedlings, using the LiCl method (Verwoerd et al., 1989). Expression analyses were performed using Power SYBR Green PCR Master Mix (Applied Biosystems). Normalization was performed using UBIQUITIN10 (UBI10) and ACTIN8 as internal standards for *Arabidopsis* and Ppa009483m/Prupe.8G137600 for peach (Primers are listed in Supplementary Table 1). qRT-PCR was performed and the obtained data manipulated as previously described (Trainotti et al., 2007).

### *In Situ* Hybridizations

*Prunus persica* S3II and S4 fruits were fixed and embedded in 4% paraformaldehyde. A *CTG134* specific probe was amplified by PCR from S3II and S4 fruit cDNAs (primers listed in Supplementary Table 1) and further cloned in pGEM T-easy vector (Promega). The CTG134 transformed vector was further used as template for the creation of sense and antisense probes by an *in vitro* transcription performed with SP6 and T7 polymerases. Sections of plant tissue were probed with digoxigenin-labeled antisense RNA-probe as previously described (Brambilla et al., 2007) and observed with a Zeiss Axiophot D1 light microscope^1^.

### pPR97-proCTG134:GUS Construct Design and GUS Assays

To assess the CTG134 promoter activity, a fragment of 2679 bp located upstream of the coding sequence initiation site (Supplementary Figure 1A) was isolated from peach genomic DNA (cv. Red Haven) by PCR. PCR product was cloned into the pCR8/GW/TOPO TA Cloning vector (Invitrogen, Carlsbad, CA, United States), according to the manufacturer’s instructions and confirmed by sequencing. The promoter fragment was thus subcloned into a pPR97-derived vector (12.20 kb), made compatible with the Gateway cloning system (LR Clonase II – Invitrogen, Carlsbad, CA, United States). This modified pPR97 vector with kanamycin resistance was employed for stable transformations both in *A. thaliana* and *N. tabacum*, to measure the CTG134 promoter activity. The promoter was tested by cloning the upstream sequence and a GUS reporter gene interrupted by a plant intron (Vancanneyt et al., 1990). To make easier the cloning, a CC_rfA gateway cassette was inserted (SmaI) upstream of the reporter gene and the antibiotic kanamycin was used to select resistant successfully transformed plants. For the GUS histochemical assay (Jefferson et al., 1987), tissues were cut and immersed into 1 mM X-Gluc (5-bromo-4-chloro-3-indolyl β-D-glucuronide), 100 mM phosphate buffer pH 8.5, 0.1% (v/v) Triton X-100, 0.5 mM K_3_Fe(CN)_6_, 0.5 mM K_4_Fe(CN)_6_, 10 mM EDTA, 20% (v/v) methanol. After a vacuum treatment of 5 min to facilitate the penetration of the dying solution, tissues were kept for 12 h in the dark at 37°C. Samples were then fixed and destained with 50% acetic acid in methanol and stored in 70% (v/v) ethanol. For the enzymatic GUS assays, proteins were extracted in 1.7 ml/g fresh weight of modified CCRL buffer (100 mmol/L K-phosphate pH 7.8, 1 mmol/L EDTA, 10% glycerol) added before use with 7 mmol/L β-mercaptoethanol and 0.1% Triton X-100, (Luehrsen et al., 1992). The homogenate was centrifuged twice for 15 min and the clear supernatant was used for either protein (Bradford, 1976) or reporter activity quantification. The GUS assay was carried out by incubating 50–200 μL of protein extract with the substrate 4-methylumbelliferyl-β-D-glucuronide (MUG) at 37°C. The released 4-methylumbelliferone (4-MU) was quantified with a Hoefer TKO 100 mini-fluorometer according to the manufacturer’s instructions. The GUS activity was expressed as nmol 4-MU released in a minute per microgram of protein.

### pGreen-AmpR-KanNos-35S:CTG134 Construct Design

The CTG134 coding sequence (524 bp) was amplified by PCR from *P. persica* (cv. Red Haven, S4I development stage) cDNA and subsequently cloned into the pCR8/GW/TOPO TA Cloning vector (Invitrogen, Carlsbad, CA, United States). The CTG134 CDS was further inserted into a pGreen-derived vector (Hellens et al., 2000) with the Gateway cloning system (LR Clonase II – Invitrogen, Carlsbad, CA, United States). The pGreen-derived vector was modified to confer resistance to both kanamycin and ampicillin. Moreover, a CC_rfA gateway cassette was inserted downstream of the 35S promoter in the EcoRV site. As before, the selection of plants was carried out with kanamycin (Supplementary Figure 1B).

### *Arabidopsis thaliana* and Tobacco Transformation

Single PCR-positive *Agrobacterium* GV3101 colonies were used to grow liquid cultures for the transformation of *A. thaliana* Columbia 0 plants with the floral dip method (Clough and Bent, 1998). The first flowers of 4-weeks old plants were cut to allow, after 4–8 days, the growing of a second set, further dipped in a suspension of *Agrobacterium* cells (OD_600_ = 0.8), sucrose (5% m/v) and Silwet L-77 (0.05%). Plants were incubated in the dark for 16 h before a second growing phase in growth chamber (16/8 light/dark cycle, 25°C, 70% relative humidity) until seeds were obtained. Transformed plants were screened on solid ½ MS medium (MS salts with vitamins 2.17 g/L, sucrose 15 g/L, pH 5.75) supplemented with kanamycin (50 mg L^-1^). After 1 week, the resistant plants were planted into soil and grown in greenhouse for at least two generations, until T-DNA insertions reached homozygosity. Plants were screened for the presence of the transgene by PCR on genomic DNA using specific primer pairs. *In vitro* grown *N. tabacum* SNN plants were instead transformed following the protocol reported by (Fisher and Guiltinan, 1995). As for *Arabidopsis*, plants were screened for the presence of the transgene with PCR on genomic DNA using specific primer pairs.

### Peptide Synthesis

The peptides DYSPARRKPPIHN and DY(SO_3_H_2_) SPARRKPPIHN were synthesized by automatic solid phase procedures. The synthesis was performed using a multiple peptide synthesizer (SyroII, MultiSynTech GmbH) on a pre-loaded Wang resin (100–200 mesh) with *N*-α-Fmoc-*N*-β-trityl-l-asparagine (Novabiochem, Bad Soden, Germany). The fluoren-9-ylmethoxycarbonyl (Fmoc) strategy (Fields and Noble, 1990) was used throughout the peptide chain assembly, utilizing *O*-(7-azabenzotriazol-1-yl)-*N*,*N*,*N*′,*N*′-tetramethyluronium hexafluorophosphate (HATU) as coupling reagent (Carpino et al., 2001). The side-chain protected amino acid building blocks used were: *N*-α-Fmoc-β-tert-butyl-l-aspartic acid, *N*-α-Fmoc-*N*ε-tert-butyloxycarbonyl-l-lysine, *N*-α-Fmoc-*N*ω-2,2,4,6,7-pentamethyl dihydrobenzofuran-5-sulfonyl-l-arginine, *N*-α-Fmoc-*O*-tert-butyl-l-serine, *N*-α-Fmoc-N(im)-trityl-l-histidine, *N*-α-Fmoc-*O*-tert-butyl-l-tyrosine and *N*-α-Fmoc-*O*-sulfo-l-tyrosine tetrabutylammonium salt. Cleavage of the peptides was performed by incubating the peptidyl resins with trifluoroacetic acid/H2O/triisopropylsilane (95%/2,5%/2,5%) for 2.5 h at 0°C. Crude peptides were purified by a preparative reverse phase HPLC. Molecular masses of the peptide were confirmed by mass spectroscopy on a MALDI TOF-TOF using a Applied Biosystems 4800 mass spectrometer.

### Ca^2+^ Measurement Assays

Ca^2+^ measurement assays were carried out in cell suspension cultures obtained from *Arabidopsis* seedlings stably expressing cytosolic aequorin (seeds kindly provided by M.R. Knight, Durham, United Kingdom). Reconstitution of aequorin and Ca^2+^ measurements were carried out as described by (Sello et al., 2016).

## Results

### Regulation of CTG134 Expression

**FIGURE 1:**
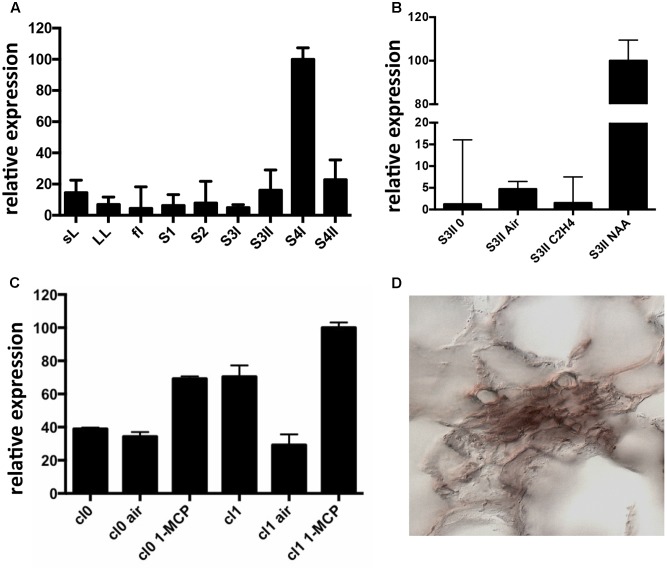
Expression profile of *CTG134*. **(A)** *CTG134* expression was barely detectable, by qRT-PCR, in non-fruit organs (small expanding leaves -sL- and fully developed leaves –LL-) and in fruit at early development (stage 1 and 2, -S1, S2-). In mature fruit (S3II) there was a sharp increase in *CTG134* transcription (S4I), slightly ceasing after the ethylene peak (S4II). **(B)** Ethylene, auxin and 1-MCP responsiveness of *CTG134. CTG134* expression, barely detectable in mature preclimacteric fruit (S3II 0) was strongly increased upon auxin (NAA, 1-naphthalene acetic acid, a synthetic auxin) but not ethylene treatment. **(C)** Both in Class 0 and Class 1 S4 fruit, 1-MCP upregulated *CGT134* expression. **(D)** Localization of *CTG134* expression in peach mesocarp by *in situ* hybridization. In peach mesocarp at S4, *CTG134* expression was mainly associated with vascular bundles (control sections in Supplementary Figure 2). Scale bar = 50 μm.

Expression of *CTG134* was assessed in peach mesocarp during the onset of fruit ripening (i.e., at early stage 4 –S4I – **Figure 1A**). CTG134 mRNA accumulated in preclimacteric fruit (i.e., S3II) after auxin treatment, while exogenous ethylene had no effect (**Figure 1B**). Moreover, treatment with the ethylene inhibitor 1-MCP induced *CTG134* transcription at stages before (cl 0) and coincident (cl 1) with the full climacteric (**Figure 1C**). The peach mesocarp at ripening is mainly made up of parenchymal cells and vascular tissue (Zanchin et al., 1994). To localize the types of cells expressing *CTG134* at ripening, *in situ* hybridization experiments were carried out with mesocarp sections prepared by peach fruit in S4 stage. The CTG134 mRNA was localized in vascular bundles (Supplementary Figure 2C), most likely in the phloem or parenchymal cells (**Figure 1D**).

**FIGURE 2:**
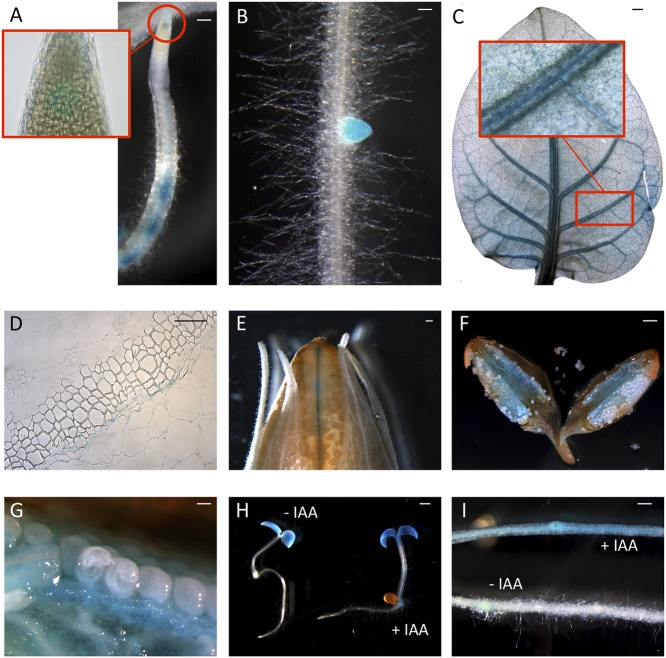
*ProCTG134:GUS* expression in tobacco and auxin responsiveness. **(A,B)** In tobacco roots the expression of GUS was detected at the level of the RAM (inset) but mainly at the level of lateral root primordia. **(C)** Staining was detectable also in leaves, especially if treated with 50 μM IAA, and particularly in veins (inset). **(D)** In the stem, GUS expression was more abundant in parenchymatic cells of the vascular tissue. In the fruit expression was visible at the dehiscence zone **(E)** and in the placenta **(F,G)**. **(H)** Auxin responsiveness in 1-week-old representative seedlings (untreated on the left, and treaded with 50 μM IAA on the right) and in the root (**I**, untreated, on the bottom, and treaded with 50 μM IAA, on the top). Scale bar in **(B,C,F)** = 500 μm, in **(A)** = 200 μm, in **(D)** = 100 μm and in **(E)** = 1000 μm.

**FIGURE 3:**
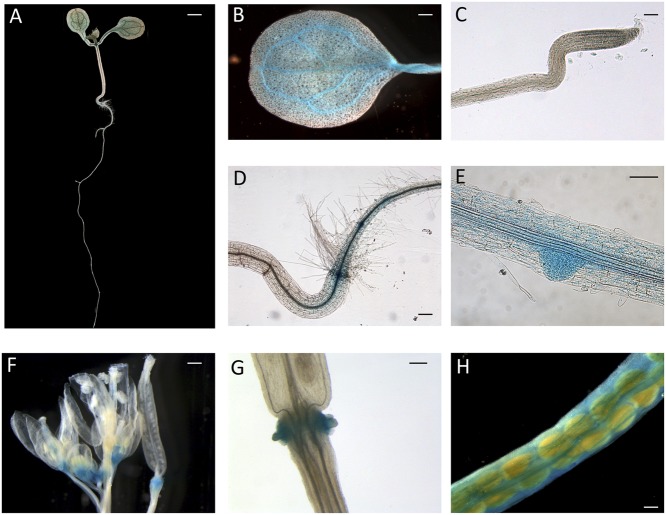
*ProCTG134:GUS* expression in *Arabidopsis*. At 7 days after germination **(A)**, GUS staining is detectable in cotyledons, especially in veins **(B)**, at the root–shoot transition zone **(D)** and in lateral root primordia **(E)**, while is barely detectable in RAM **(C)**. In the reproductive part, expression was detected in abscission zones before **(F)** and after **(G)** organ shedding. Expression was detectable also in maturing siliques mainly associated with vascular bundles **(H)**. Scale bar in **(B,C,F)** = 500 μm, in **(A)** = 200 μm, in **(D)** = 100 μm and in **(E)** = 1000 μm.

Since peach is a recalcitrant species to transform, pro*CTG134:GUS* lines were generated in both tobacco and *Arabidopsis* model species. In tobacco, a slight but evident GUS staining was detected in the apical meristem (RAM) of *in vitro* grown lateral roots (**Figure 2A**). Moreover, a dark staining was visible in lateral root emergence (**Figure 2B**) as well as in leaf, mainly associated, but not limited to, the vascular tissue (**Figure 2C**). In the stem of 1-week-old plantlets, GUS expression was localized in phloem of cell layers closed to the cambium (**Figure 2D**). GUS expression was also tested in reproductive organs, where it was detected in the tips of both young sepals and petals (not shown) and in capsules at the level of the dehiscence zone (**Figure 2E**). The inner part of the fruit was the part more significantly stained (**Figures 2F,G**), with the highest expression in the placenta (**Figure 2G**). On the contrary, in all the transgenic lines investigated in this study, the GUS coloration was never observed in ovule. In 1-week-old tobacco seedlings the reporter was more expressed in cotyledons than roots. However, 5-h treatment with 50 μM IAA induced a different GUS staining in the entire shoot apex and root, reaching the highest intensity in the root-stem transition zone (**Figure 2H**). A similar auxin-induced expression was also observed in roots of *in vitro* grown plantlets (**Figure 2I**). The stimulation of the GUS staining in tobacco finds also consistency with the aforementioned expression pattern of CTG134 in peach fruit. The expression of this element was in fact enhanced by auxin (**Figure 1B**) and auxin responsive elements (AREs) were moreover detected in the *CTG134* promoter region (Supplementary Figure 1B). To further validate the heterologous analysis carried out in tobacco, the activity of the CTG134 promoter was additionally investigated in *Arabidopsis* (**Figure 3A**). Also in this species, the GUS expression was higher in cotyledons (**Figure 3B**) rather than in primary root, where the GUS staining was undetectable in the RAM (**Figure 3C**). The GUS activity was instead clearly visible at the root-stem transition zone (**Figure 3D**) and during lateral root emergence (**Figure 3E**). In the reproductive organs, the expression pattern was detected in abscission zones before (**Figure 3F**) and after (**Figure 3G**) shedding. The expression was also detected in maturing siliques and leaves, especially in those associated with vascular bundles (**Figure 3H**).

### Hormonal Regulation of CTG134 in Tobacco

**FIGURE 4:**
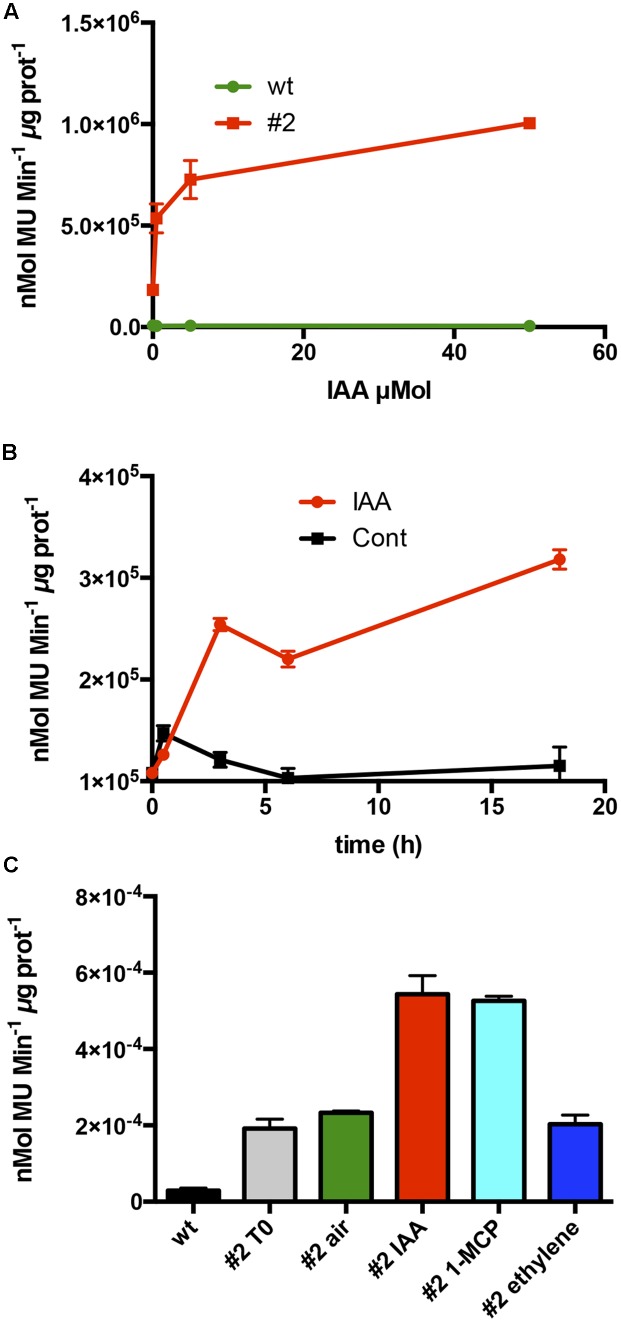
Hormone responsiveness of *ProCTG134:GUS* in tobacco seedlings. **(A)** Auxin was effective in inducing the promoter of *CTG134* already at 0.5 μM, to reach almost complete saturation at 50 μM. **(B)** Saturation of the auxin induction after 3 h. **(C)** Besides auxin, also 1-MCP had an inductive effect on the promoter of *CTG134*, while ethylene seemed ineffective. All experiments were carried out with T3 seedlings of line #2.

To test whether the auxin responsiveness was due to the promoter regulatory region, 1-week old tobacco seedlings of line #2 were exposed to increasing concentrations of IAA. The *CTG134* promoter was responsive to IAA already at 0.5 μM, with an activity pattern proportional to the hormone concentrations. The system reached saturation at 50 μM (**Figure 4A**). The IAA induction kinetic was assessed over a time course of 20 h on tobacco seedlings of line #2 treated with 10 μM IAA. An initial slight induction in both control and treated samples was observed already after 30 min, after which the GUS activity remained at a basal level in the control, while in the IAA treated samples a significant burst was observed after 3 h after the treatment (**Figure 4B**). Since in peach fruit the expression of *CTG134* was insensitive to ethylene and induced by 1-MCP (**Figures 2B,C**), the promoter responsiveness was tested by treating 10-day-old tobacco seedlings for 16 h with ethylene (10 μL L^-1^), IAA (10 μM) and 1-MCP (1 μL L^-1^). 1-MCP induced the reporter activity similarly to auxin (**Figure 4C**), while treatment with ethylene did not change the expression of the GUS reporter gene.

### Over-Expression of *CTG134* in Tobacco

**FIGURE 5:**
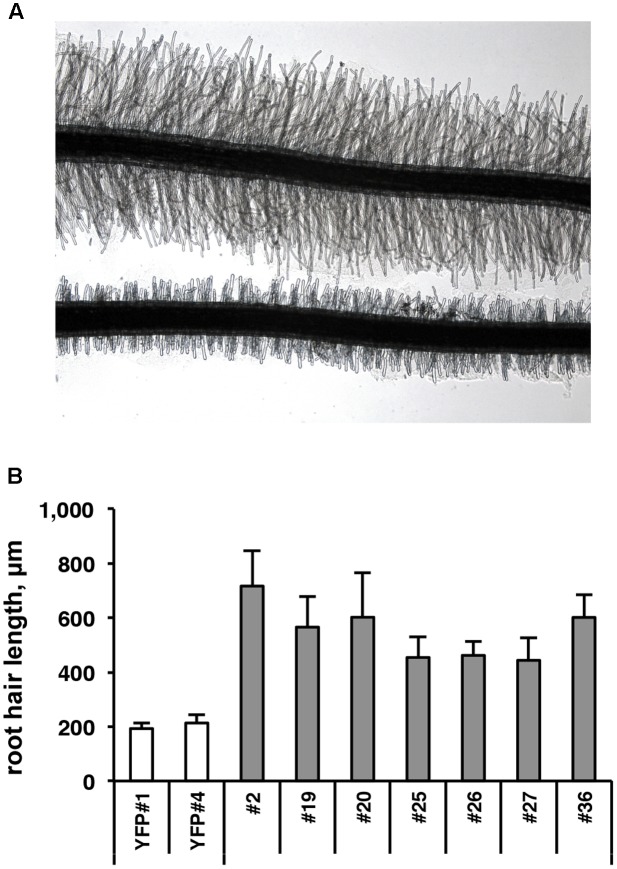
Effects on root growth of *CTG134* overexpression in tobacco. **(A,B)** CTG134 increases hair length (ANOVA, *F* = 87.75, *df* = 155, *p* < 0.001) in tobacco plantlets grown on agar (controls are transgenic plants expressing the YFP reporter, on bottom in **A**).

**FIGURE 6:**
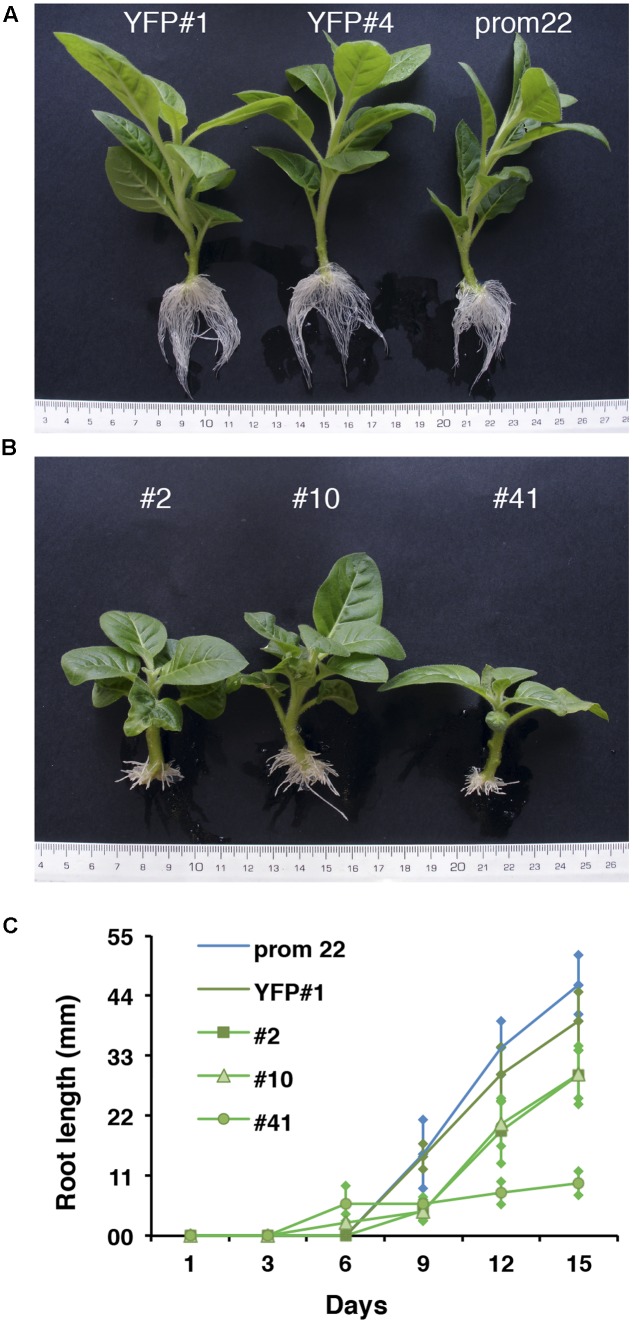
Effects on adventitious root formation of *CTG134* overexpression in tobacco. **(A)** control lines; **(B)** 35S:CTG134 tobacco clones. Controls were YFP expressing clones (YFP#1 and YFP#4) and ProCTG134:GUS line (prom 22). **(C)** CTG134 overexpression caused faster development of adventitious roots but inhibited their growth.

**FIGURE 7:**
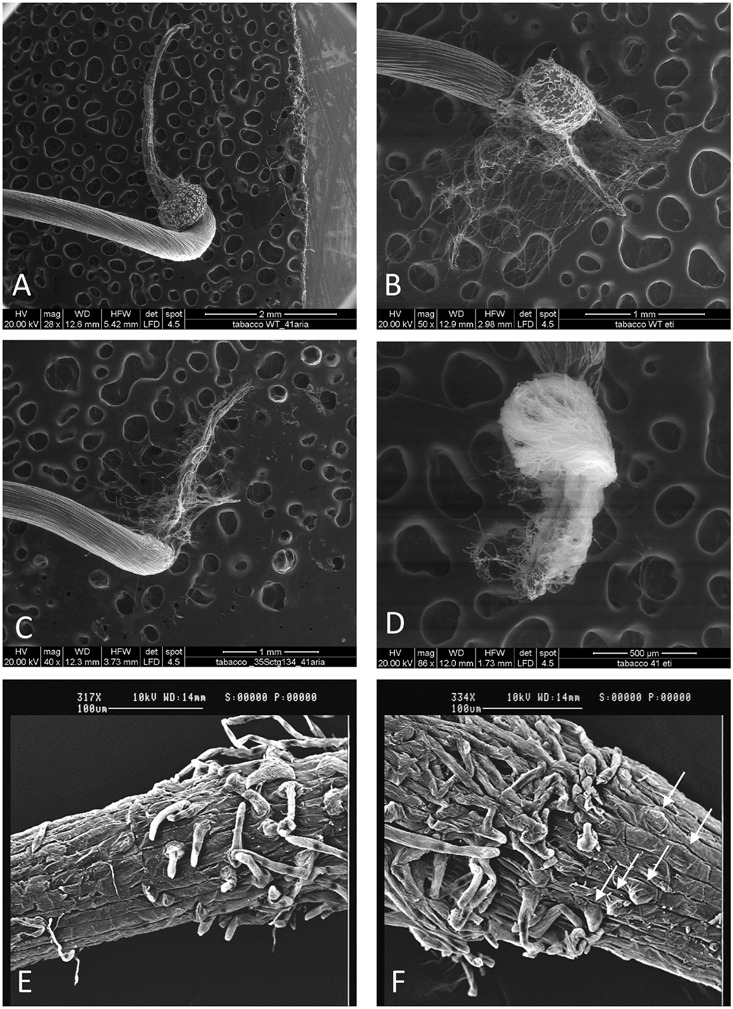
Effects on root growth of *CTG134* overexpression in tobacco seedlings. *CTG134* overexpression in tobacco did not saturate ethylene effect on root hair development and changed the developmental fate of epidermal cells. WT **(A,B,E)** and *35S:CTG134* **(C,D,F)** seedling roots were imaged by ESEM after growth in air **(A,C)** or ethylene **(B,D)**. SEM images of the transition zones of tobacco etiolated seedling roots grown in air showed trichoblasts and atrichoblasts in the WT **(E)** while almost all epidermal cells were trichoblasts in *35S:CTG134* plants (**F**; white arrows indicate the presence of root hair primordia that are emerging from epidermal cells).

To functionally investigate the role of the CTG134 peptide, its full-length coding sequence, under the control of the Cauliflower mosaic virus 35S (35S CaMV) promoter was expressed in tobacco. The development of longer root hairs was noticed already in the early phases of transgenic plant production (**Figure 5A**). A YFP gene, cloned in the same binary vector as CTG134, was overexpressed to have control plants able to grow on kanamycin and gentamicin present in the growth media. To further assess this phenotype, scions from different clones were propagated and primary roots from 30 day-old plants were analyzed by taking images in the root portion located at 6 mm from the root tip. On average, the CTG134 overexpressing lines showed an increase of at least twofold in root hair length (ANOVA, *F* = 87.75, *df* = 155, *p* < 0.001) with respect to control wild type plants (**Figure 5B**). The effect on root development was also evident during adventitious roots formation in *in vitro* plants (**Figures 6A,B**). Indeed, root primordia emerged earlier in *35S:CTG134* scions than in controls, although the root growth was slower, resulting at the end in shorter roots (**Figure 6C**). Within the hypothesis of the auxin-ethylene crosstalk, the putative mediating role of CTG134 was investigated exposing to ethylene (10 μL L^-1^) 35S:CTG134 transformed tobacco seedlings grown in the dark. Environmental Scanning Electron Microscopy (ESEM, **Figures 7A–D**), confirmed the difference in the root hair phenotype, but a clear distinction between transgenic lines and controls for the apical hook and hypocotyl thickening, typical of the triple ethylene response, was not observed. Indeed, the untreated (air) 35S:CTG134 (**Figure 7C**) seedlings displayed a phenotype similar to controls grown in presence of ethylene (**Figure 7B**), despite the fact that samples were partially dehydrated by the light vacuum imposed during the ESEM observation. Interestingly, the ethylene treatment induced an additional phenotype in the 35S:CTG134 lines, provoking the development of a massive root hair formation, completely wrapping the root body (**Figure 7D**). Scanning Electron Microscopy (SEM) analyses disclosed that the previously observed root hair phenotype was due not only to an increase of their length but also of their density in the 35S:CTG134 lines (**Figure 7E**) compared to control (**Figure 7F**). Indeed, most of the root epidermal cells of 35S:CTG134 seedlings developed root hairs, while in WT trichoblasts were arranged in alternating files with atrichoblasts along the root surface.

**FIGURE 8:**
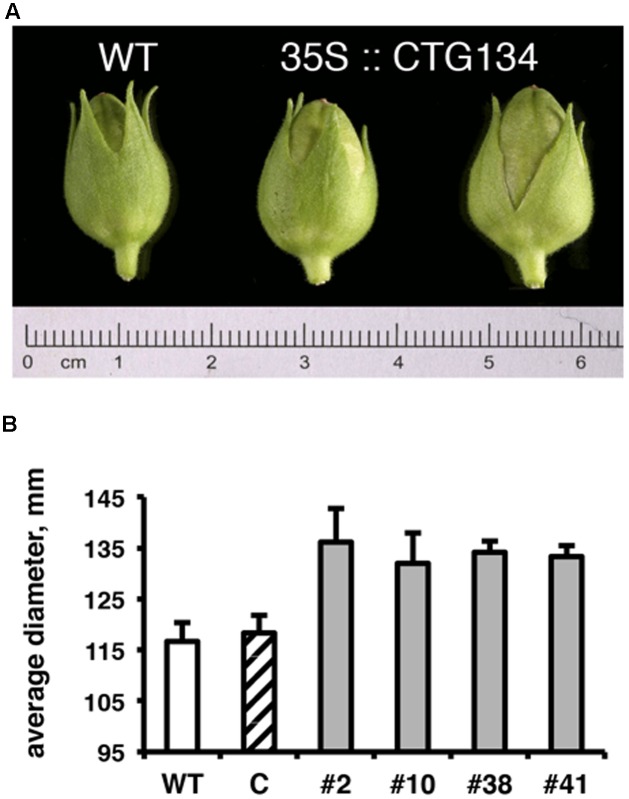
Effects on capsule size of *CTG134* overexpression in tobacco. **(A)** Representative capsules collected from tobacco plants 12 days post-anthesis. **(B)** The capsule diameter of *CTG134* overexpressing lines was larger than that of WT or control (*ProCTG134:GUS*) transgenic lines.

Since the CTG134 sequence was originally isolated from peach fruit, and placenta cells were stained in tobacco plants expressing the GUS reporter gene driven by the CTG134 promoter, tobacco transgenic capsules were also analyzed. Even if tobacco produces a dry fruit structurally different from the fleshy stone fruit of peach, the CTG134 overexpression led to a detectable increase in fruit size. Tobacco capsules of 35S:CTG134, harvested 12 days after anthesis (before drying), showed an increase in diameter of about 16% with respect to wild type or 35S:YFP (ANOVA, *F* = 3,85, *df* = 22, *p* = 0.013) (**Figure 8**).

### Over-Expression of *CTG134* in *Arabidopsis*

**FIGURE 9:**
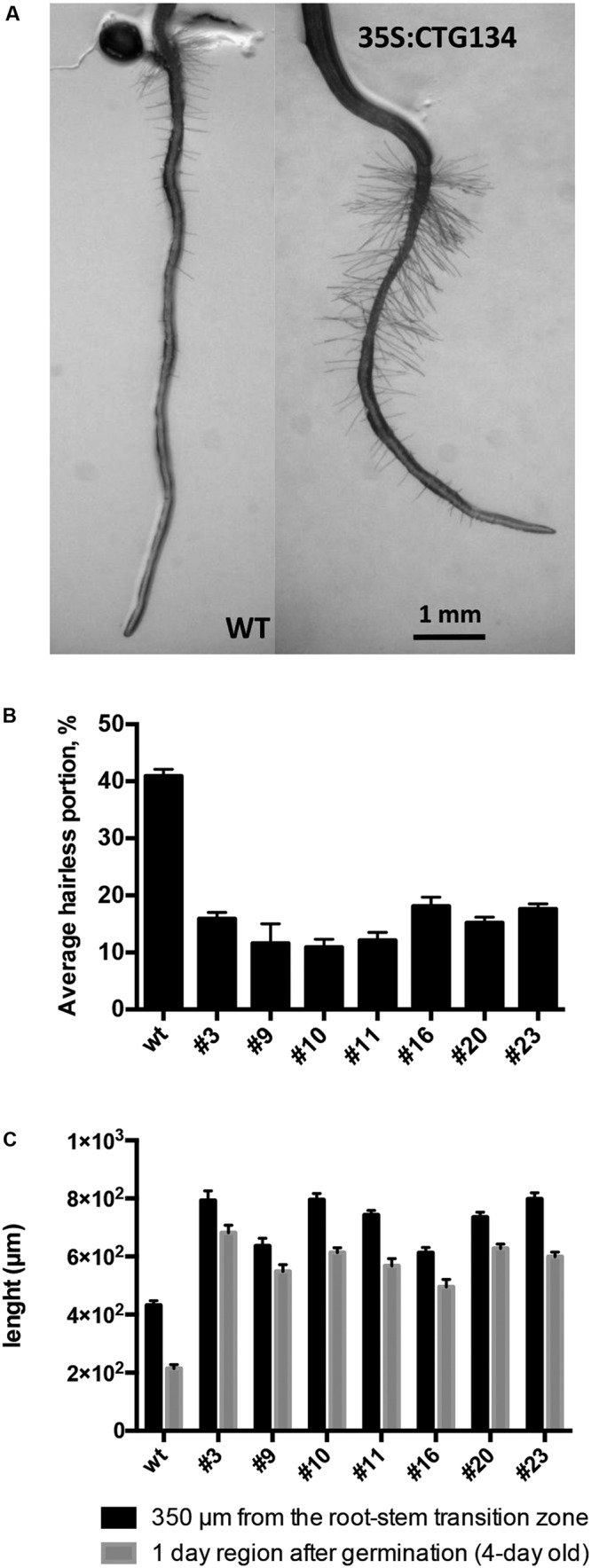
Effects on root development of *CTG134* overexpression in *Arabidopsis*. CTG134 increased hair length (Measurements at 350 μm from the root-stem transition zone: ANOVA, *F* = 95.07, *df* = 342, *p* < 0.001; Measurements at 1 day region after germination: ANOVA, *F* = 98.31, *df* = 342, *p* < 0.001) in *Arabidopsis* plantlets grown on agar **(A,C)**; moreover, the portion without root hairs was reduced (ANOVA, *F* = 101.1, *df* = 23, *p* < 0.001) **(A,B)**.

**FIGURE 10:**
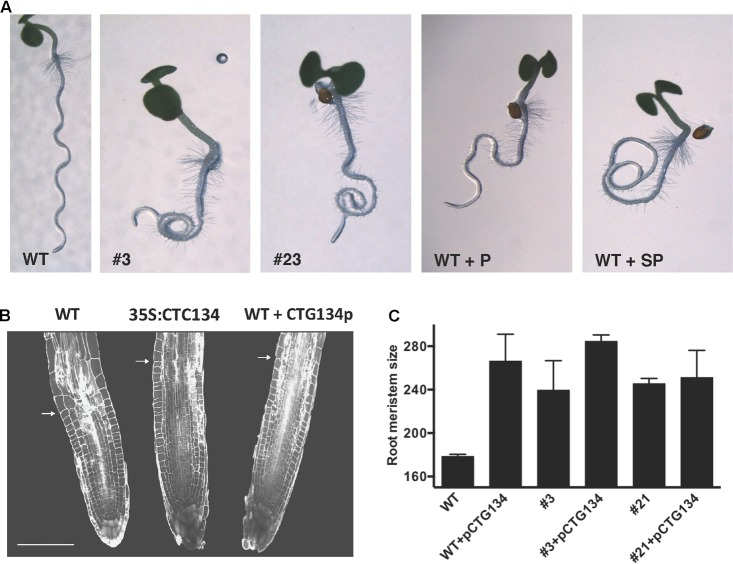
Effects of CTG134 on root gravity perception and on meristem size in *Arabidopsis*. **(A)** WT seedlings grown on oblique agar plates showed roots with a regular wavy patter that was altered in CTG134 overexpressing lines (**A**, #3 and #23). Alteration of the wavy pattern was observed also on WT seedlings grown with synthetic CTG134 peptide added to the medium. The effect was stronger if the added peptide was tyrosine-sulfated (WT+SP) compared to the non-sulfated form (WT+P). **(B)** *Arabidopsis* root sections at five DAG, stained with propidium iodide (WT, 35S:CTG134 = overexpressing line, WT + CTG134p = WT grown in the presence of a tyrosine-sulfated synthetic CTG134 peptide). White arrows indicate the transition zone. Scale bar = 100 μm. **(C)** Measures of meristem size were statistically (Tukey’s multiple comparisons test) larger in comparisons among WT and overexpressing lines (#3 and #21), WT grown in the presence of a tyrosine-sulfated synthetic CTG134 peptide (WT+pCTG134) and overexpressing lines grown in the presence of a tyrosine-sulfated synthetic CTG134 peptide (#3+pCTG134 and #21+pCTG134). Meristem sizes were not statistically different if WT was excluded. Root meristem was measured using ImageJ software.

Similarly to tobacco, the same construct was further employed to transform *Arabidopsis*. T2 CTG134 overexpressing lines were easily identified for their root phenotype when grown on horizontal plates. The primary root of 5-day-old 35S:CTG134 seedlings had indeed longer hairs than WT ones (**Figure 9A**). Moreover, root hairs developed closer to the apex that in WT roots. Consequently, the hairless portion of the root was about half (ANOVA, *F* = 101.1, *df* = 23, *p* < 0.001) of that in the WT (**Figure 9B**). As regards to root hair length, being not uniform along the root and clearly depending on age, sizes were taken at given distances from the root-stem transition zone and in a region of the tip that was determined to be, based on growth rate, 4-day old. Both measures clearly indicated that the root hairs in the overexpressing lines were longer (ANOVA, *F* = 95.07, *df* = 342, *p* < 0.001; ANOVA, *F* = 98.31, *df* = 342, *p* < 0.001, respectively) than wild type (**Figure 9C**). Members of the RGF/GLV family in *Arabidopsis* are known to induce developmental defects in roots when over-expressing seedlings were grown on tilted plates (Whitford et al., 2012; Fernandez et al., 2013). Accordingly, in this work *Arabidopsis* 35S:CTG134 seedlings produced roots with larger and more irregular waves than the WT (**Figure 10A**). This effect could be phenocopied by the WT when the synthetic CTG134 peptide (pCGT134) was added to the medium, with the sulfated form being more active than the non-sulfated one (**Figure 10A**). Albeit the hairless portion of the root was shorter in overexpressing seedlings, the meristematic region of the root was longer. Moreover, both 35S:CTG134 lines and WT seedlings grown in a medium supplemented with pCTG134 had an increase in root meristem size (**Figures 10B,C**). The effect on the root meristem size was saturable, as overexpressing lines did not respond to exogenous pCTG134 as the WT (**Figure 10C**).

**FIGURE 11:**
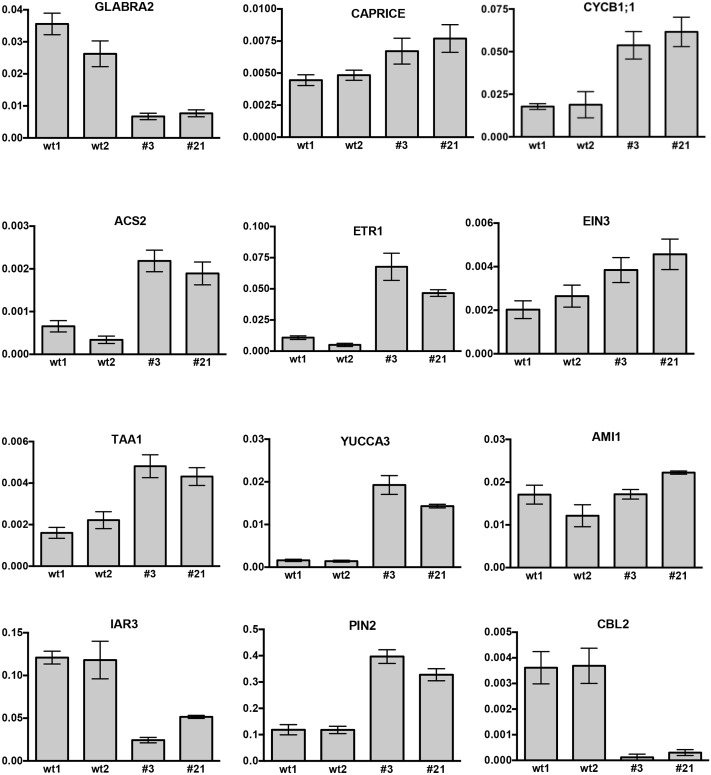
Relative expression profiles of selected genes in roots of *Arabidopsis* seedlings grown on agar plates for 5 days. wt1 and wt2 are wild type samples collected from two different plates, while #3 and #21 are the clone identifiers of the *Arabidopsis* lines overexpressing the peach *CTG134* gene. Values (means of the normalized expression) have been obtained by real-time qRT-PCR analyses. Bars are the standard deviations from the means of three replicates. ACT8 was used as reference gene.

The effect of *CTG134* overexpression at the transcriptional level was tested on 5-day-old seedling roots (**Figure 11**). Alteration in root hairs morphology and quantity was accompanied with a reduction of *GLABRA2* (*GL2*) and a slight induction of *CAPRICE* (*CPC*) expression. The increased meristem size was supported by the expression of *CYCLIN B1;1* (*CYCB1;1*). The development of root hair was selected as a suitable developmental process to test the effect of CTG134 on the interactions between ethylene and auxin occurring at the onset of peach ripening, since the crosstalk of the two hormones during root hair development is well documented (reviewed by Van de Poel et al., 2015). The expression of the ethylene biosynthetic gene *ACS2* was induced in roots of 35S:CTG134 seedlings (**Figure 11**), as well as that of *ETR1* and *EIN3*, encoding an ethylene receptor and a transcription factors starting the transcriptional cascade leading to ethylene responses, respectively. On the contrary, transcription of *CTR1*, encoding the first downstream signaling component after the ethylene receptor(s) (Kieber et al., 1993) was unaffected (Supplementary Figure 3). About auxin, both *TAA1* and *YUC3* and *6* genes involved in the indole-3-pyruvic acid branch of the hormone synthesis pathway (Tivendale et al., 2014) were induced in *CTG134* overexpressing seedlings, while *AMI1*, involved in the indole-3-acetamide branch of the pathway, seemed unaffected (**Figure 11** and Supplementary Figure 3). Free auxin levels depend not only on hormone synthesis but also on its release from storage compartments and transport. The expression of *IAR3*, a gene encoding an IAA-Ala hydrolase (Davies et al., 1999), decreased in *CTG134* overexpressing plants, while *PIN2*, encoding an auxin efflux carrier (Müller et al., 1998) was induced (**Figure 11**).

### pCTG134 Induces a Cytosolic Ca^2+^ Change

In peach, a gene encoding a Ca^2+^ sensing protein belonging to the Calcineurin B-like (CBL) family (*CTG85*) mirrored the expression of *CTG134* during fruit ripening, as well as after 1-MCP treatment (Tadiello et al., 2016). The expression of *CBL1*, *2*, *4*, and *10* encoding genes was therefore tested in 35S:CTG134 roots, showing a general repression, with *CBL2* as the most severely down-regulated gene (**Figure 11** and Supplementary Figure 3).

**FIGURE 12:**
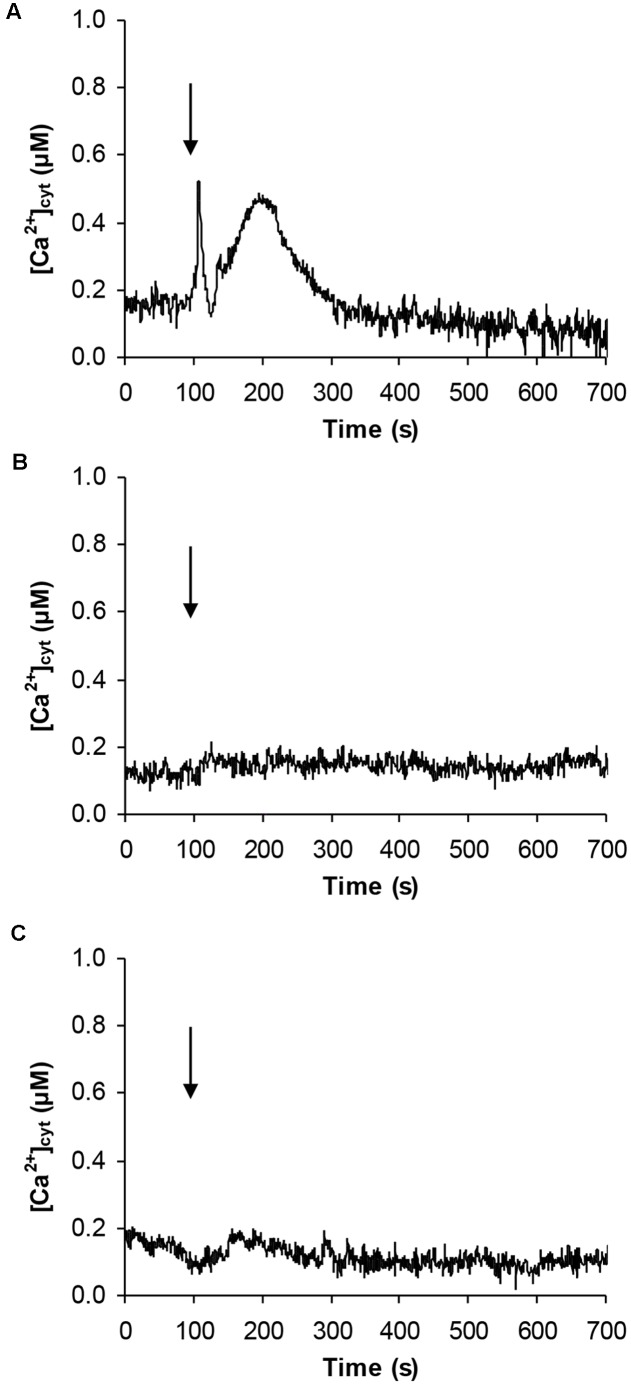
Induction of a transient cytosolic Ca^2+^ change by the sulphonated peptide CTG134S in *Arabidopsis*. Cytosolic Ca^2+^ concentration ([Ca^2+^]_cyt_) was monitored in aequorin-expressing *Arabidopsis* cell cultures in response to 100 μM CTG134S **(A)**. As controls, cells were challenged with plant cell culture medium **(B)** or with the non-specific peptide T16E S19A2 (100 μM, **C**). The arrow indicates the time of injection (100 s). Ca^2+^ traces are representative of three independent experiments which gave very similar results.

Given the effect on CBL gene expression and the potential involvement of Ca^2+^ in the signaling pathway activated by signaling peptides (Ma et al., 2013), *Arabidopsis* cell suspension cultures stably expressing the bioluminescent Ca^2+^ reporter aequorin in the cytosol were challenged with 100 μM pCTG134. Ca^2+^ measurement assays demonstrated the induction of a biphasic cytosolic Ca^2+^ transient, characterized by a rapid rise, which equally quickly dissipated, followed by a slower Ca^2+^ increase, peaking at about 0.5 μM after 100 s and falling back to basal levels within 5 min (**Figure 12A**). No changes in cytosolic Ca^2+^ concentration ([Ca^2+^]_cyt_) were detected in response to either plant cell culture medium (**Figure 12B**) or a non-specific peptide (100 μM T16E S19A2) (**Figure 12C**), supporting the specificity of the observed Ca^2+^ response to the sulfated CTG134 peptide.

## Discussion

Peptide hormones participate in both proximal and distal cell-to-cell communication processes necessary during growth as well as to cope with biotic and abiotic stimuli (reviewed in Matsubayashi, 2014; Tavormina et al., 2015; Wang et al., 2016). Despite the growing interest in peptide hormones, their possible role during fleshy fruit ripening remains almost unexplored (Zhang et al., 2014). In peach fruit, gene expression profiling suggested that *CTG134*, encoding a peptide belonging to the RGF/GLV family, could be involved in the crosstalk between auxin and ethylene occurring at the onset of fruit ripening (Tadiello et al., 2016).

### CTG134 Expression Is Ripening Specific and Affected by Auxin and Ethylene Perception

Extensive RNA profiling confirmed that *CTG134* is expressed almost exclusively at the onset of ripening, during the transition stage from system 1 to 2 (**Figure 1**), as initially suggested (Tadiello et al., 2016).

Considering the difficulties to perform functional studies in *Prunus* species due to low efficiency and slowness of their transformation, tobacco and *Arabidopsis* transgenic lines expressing the GUS reporter gene driven by the CTG134 promoter sequence, were created. The *cis*-regulatory elements present in the peach *CTG134* promoter drive *GUS* gene expression in cell/tissue types where the crosstalk between auxin and ethylene was described both in tobacco (**Figure 2**) and *Arabidopsis* (**Figure 3**). These comprise both cells undergoing separation processes, like abscission, dehiscence zones, lateral root primordia (Roberts et al., 2002; Kumpf et al., 2013), cambium associated cells (Love et al., 2009; Sanchez et al., 2012) and placenta cells (De Martinis and Mariani, 1999; Pattison et al., 2015). The specificity of the GUS staining pattern obtained in heterologous systems was validated by *in situ* hybridization in peach mesocarp, where *CTG134* expression was more abundant in bundle associated cells (**Figure 1D**). It is noteworthy that also regulatory regions of tomato (Blume and Grierson, 1997), apple (Atkinson et al., 1998) and peach (Moon and Callahan, 2004) *ACO* genes drove GUS expression more abundantly in bundle than parenchyma cells of tomato pericarp. Besides spatial regulation, also hormone responsiveness within *CTG134* regulatory regions supported the role in the crosstalk between auxin and ethylene (**Figures 1**, **4**). Indeed, both on ripening mesocarp and tobacco seedlings, not only IAA had an inductive effect, probably due to the presence of AREs, but also the altered perception of ethylene (due to 1-MCP treatment) stimulated both *CTG134* transcription in ripening fruit and *GUS* accumulation in tobacco seedlings. In ripening peaches 1-MCP induced auxin synthesis (Tadiello et al., 2016), and this might be the reason of the *CTG134* induction. 1-MCP treatment might have induced IAA synthesis, and thus GUS expression, also in tobacco seedlings. In roots of *Arabidopsis* treated with silver [also blocking the perception of ethylene; (Negi et al., 2008)] the exogenous application of 1-MCP might have altered the distribution of IAA, leading to *GUS* induction.

### 35S:CTG134 Plants Show Phenotypes Related to Auxin and Ethylene Action

When *CTG134* was permanently overexpressed in tobacco and *Arabidopsis* plants (**Figures 5**–**10**), the most striking effect was related to the length and number of root hairs, mimicking the effect of exogenous treatments with auxin or ethylene (Pitts et al., 1998). Adventitious root formation and elongation in tobacco were also affected, as well as capsule size, further supporting the interplay between auxin and ethylene actions. Besides the well-known effect on root hair number and morphology reported for RGF/GLV/CLEL (Whitford et al., 2012; Fernandez et al., 2013) and CLE peptides (Fiers et al., 2005), CTG134 had an impact also on tobacco capsule size. In fact, at maturity, tobacco capsules were 16% larger than WT on average, similarly to carnation flowers treated with ethylene (Nichols, 1976). Ethylene synthesis is necessary for normal ovule development which impacts flower size (De Martinis and Mariani, 1999). The GUS staining in tobacco placenta and the larger capsules in CTG134 overexpressing plants allow therefore to hypothesize that CTG134 may corroborate auxin inductive and ethylene repressive actions during fruit setting (Martínez et al., 2013; Shinozaki et al., 2015).

### Molecular Targets of CTG134

The *Arabidopsis* root model was moreover exploited to gain insights into possible regulatory circuits associating CTG134 with auxin and ethylene (**Figures 9**–**11**). The wavy root phenotype and the increase in meristem size were observed in both overexpressing and peptide treated seedlings, confirming previous findings (Matsuzaki et al., 2010; Whitford et al., 2012). The observed increase in the meristem size was also supported by the induced expression of *CYCB1;1* (**Figure 11**), while the down-regulation of *GL2* was in agreement with its repressing role in root hair development (Ishida et al., 2008). More interestingly, genes of both auxin and ethylene synthesis, transport and transduction pathways were upregulated in CTG134 overexpressing roots, assigning to this RGF/GLV peptide a role in the auxin/ethylene crosstalk (Stepanova et al., 2007). Although we did not carry out a detailed analysis on the effects caused by the local application of CTG134 peptide (that in *Arabidopsis* controlled PIN2 abundance in the root meristem by a post-transcriptional mechanism, thus guiding auxin distribution; Whitford et al., 2012), we showed that the heterologous overexpression of the peach CTG134 peptide could be sensed in the portion of the root where receptors initiate the signaling cascade (Ou et al., 2016; Shinohara et al., 2016; Song et al., 2016). As for Peps signaling in *Arabidopsis* (Ma et al., 2013), aequorin-based Ca^2+^ measurement assays (**Figure 12**) demonstrated the induction by the sulfated peptide CTG134 of a remarkable cytosolic Ca^2+^ change, suggesting the likely involvement of Ca^2+^ as intracellular messenger in the transduction pathway activated by this signal peptide. The role of Ca^2+^ is supported also by the downregulation of several CALCINEURIN B-LIKE PROTEIN (CBL) genes in roots of CTG134 overexpressing seedlings, in agreement with the downregulation of a CBL gene in 1-MCP-treated peaches (Tadiello et al., 2016). Sensing the peptide had also the effect to induce the transcription of key genes of ethylene and auxin biosynthesis pathways and thus, reasonably, the levels of these two hormones which, ultimately, led to the observed phenotypes. While the response in the ethylene pathway is somewhat straightforward investigating the induction of key genes in its synthesis (*ACS2*), perception (*ETR1*) and signal transduction (*EIN3*), the action on the auxin pathway is more intricate. Indeed, while the increased transcription of *TAA1*, *YUC3*, and *YUC6* sustains the induction of the two-step IPA pathway, the unchanged levels of *AMI1* seemed to exclude the conversion of indole-3-acetamide (IAM) to IAA (Enders and Strader, 2015). Moreover, although only *IAR3* was tested, the contribution of conjugated forms of IAA (Sanchez Carranza et al., 2016) seemed negligible in *Arabidopsis*, while the expression of its peach homolog *CTG475* was supposed to participate to the free auxin increase measured before the climacteric production of ethylene in peach (Tadiello et al., 2016), thus complementing the role of *PpYUC11* (Pan et al., 2015). However, the induced transcription of *PIN* genes in overexpressing *Arabidopsis* seedlings (**Figure 11**) and in climacteric peaches (Tadiello et al., 2016) supported a key role of these peptides in regulating auxin distribution (Whitford et al., 2012).

### A Role for CTG134 As Mediator in the Auxin/Ethylene Crosstalk

**FIGURE 13:**
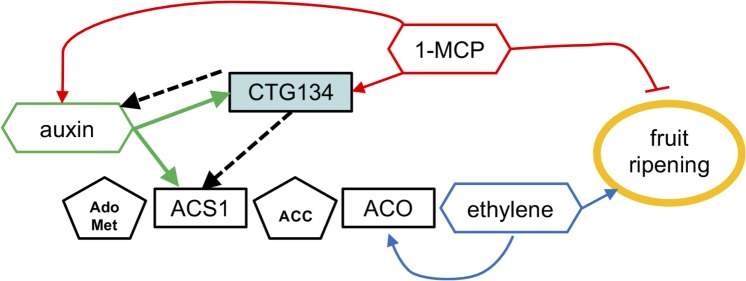
A model positioning *CTG134* in the regulatory network controlling peach ripening. Regulatory data collected from the *Arabidopsis* CTG134 overexpressing clones are represented by dashed lines. Ethylene autocatalytic synthesis and action on fruit ripening is represented in blue, auxin, 1-MCP and CTG134 interactions in green, red and black, respectively. Hormones (or inhibitors) are in hexagons, their precursors in pentagons while genes (gene products) are in rectangles. Filled arrow means induction, while blunted lines repression.

The comprehensive expression profiling data carried out in peach (Tadiello et al., 2016) and the knowledge here achieved about CTG134 in tobacco and *Arabidopsis* provide evidence on the involvement of this RGF/GVL secreted peptide in a regulatory circuit that sustains auxin and ethylene actions. The same circuit, working in both rosids (*Arabidopsis*) and asterids (tobacco) might have appeared early during evolution of eudicots to participate in the control of root hair development and later it could have been recruited in peach to regulate the switch from system 1 to system 2 ethylene synthesis (**Figure 13**). Further research will be necessary to clarify the molecular details by which CTG134 acts to either regulate auxin and ethylene synthesis or modify their distribution and perception, or both. The kinase nature of GLVs receptors (Ou et al., 2016; Shinohara et al., 2016; Song et al., 2016) agrees with the measured Ca^2+^ perturbations.

The unique mechanism that switches ethylene synthesis from system 1 to system 2 in peach probably relies on the use of a single ACS gene for both kinds of syntheses (Tadiello et al., 2016), thus differing from tomato (Barry et al., 2000) and apple (Wang et al., 2009). In these two latter fruits, the expression of *LeACS4* and *MdACS3* (system 1) is necessary to start *LeACS2* and *MdACS1* transcription (system 2), respectively. During peach ripening, expression of other ACS genes is, if present, several orders of magnitude lower than that of *ACS1* (Tadiello et al., 2016). The different amount of ethylene released by system 1 and system 2 could be achieved by modulating system 1 ACS1 activity, thus leading to system 2 *ACS1* increased transcription. ACS1 belongs to type-1 ACS proteins, which are stabilized by phosphorylation mediated by mitogen-activated protein kinases (MAPKs) (Liu and Zhang, 2004). Phosphorylation cascades have been shown to start upon binding of peptide signals (e.g., IDA) with their receptors (e.g., HAE/HSL2) (Cho et al., 2008). Given the transcriptional regulation of *CTG134*, the nature of pCTG134 and of the *Arabidopsis* receptors of its homologous RGF/GLV peptides (Ou et al., 2016; Shinohara et al., 2016; Song et al., 2016) and of the ability of pCTG134 to trigger a cytosolic Ca^2+^ signal, we hypothesized that the transition of ethylene synthesis from system 1 to system 2 in peach could be controlled by ACS1, whose activity might be therefore modulated through the action of pCTG134. The apoplastic nature of these ligands and the possibility to modulate their biological activity by antagonistic forms (Lee et al., 2015) open the possibility for the rational design of novel and environmental friendly agrochemicals with the potential of being used both in the field and during post-harvest to improve fruit storage and fruit quality in a rapidly changing environment.

## Author Contributions

Conceptualization: LT. Methodology: NB, DM, LN, FR, and LT. Investigation: NB, LN, SQ, FR, and US. Writing – Original Draft: NB and LT. Writing – Review and Editing, NB, LN, US, FC, and LT. Funding Acquisition: LT. Resources: DM, OM, and MP. Supervision: LT and DM.

## Conflict of Interest Statement

The authors declare that the research was conducted in the absence of any commercial or financial relationships that could be construed as a potential conflict of interest.

## Footnotes

1 http://www.zeiss.com
