# Supplementary material for: The Peach RGF/GLV Signaling Peptide pCTG134 Is Involved in a Regulatory Circuit That Sustains Auxin and Ethylene Actions
Source: Front Plant Sci. 2017 Oct 11;8:1711. doi: 10.3389/fpls.2017.01711 (PMC5641559; doi:10.3389/fpls.2017.01711)
Supplement: Supplementary file 1 [file Image_1.PDF]

Supplementary Material

The peach RGF/GLV signalling peptide pCTG134 is involved in a regulatory circuit that sustains auxin and ethylene actions

Nicola Busatto[1], Umberto Salvagnin[1], Francesca Resentini[1], Silvia Quaresimin[1], Lorella Navazio[1], Oriano Marin[2], Maria Pellegrini[3], Fabrizio Costa[4], Dale F. Mierke[3], Livio Trainotti[1*]*

[*]**Correspondence:** Livio Trainotti, livio.trainotti@unipd.it

Supplementary Figures

A

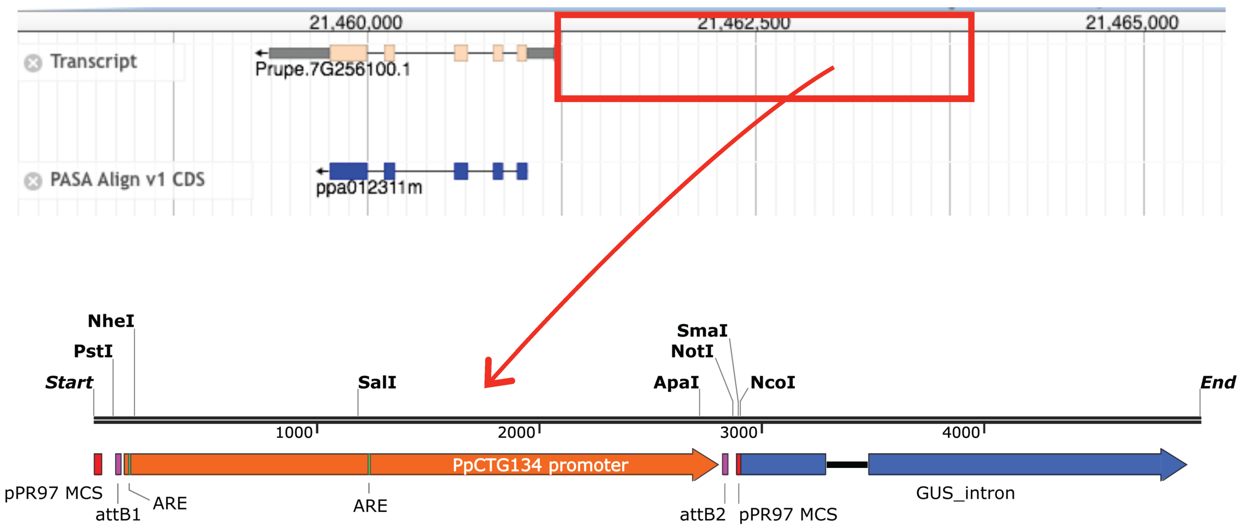

B

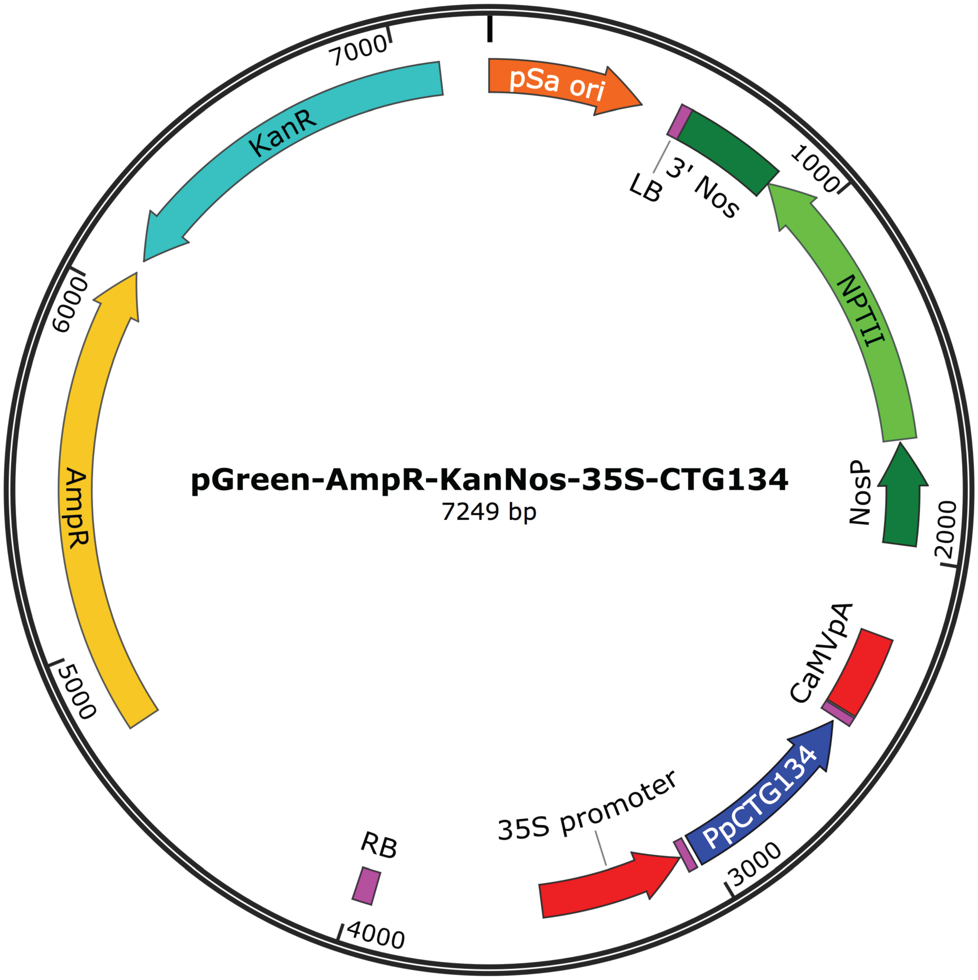

Supplementary Figure 1. Details of the vectors used. (A) T-DNA used in pPR97 for CTG134 promoter analysis. (B) Map of pGreen-AmpR-KanNos-35S-ctg134 used for CTG134 overexpression.

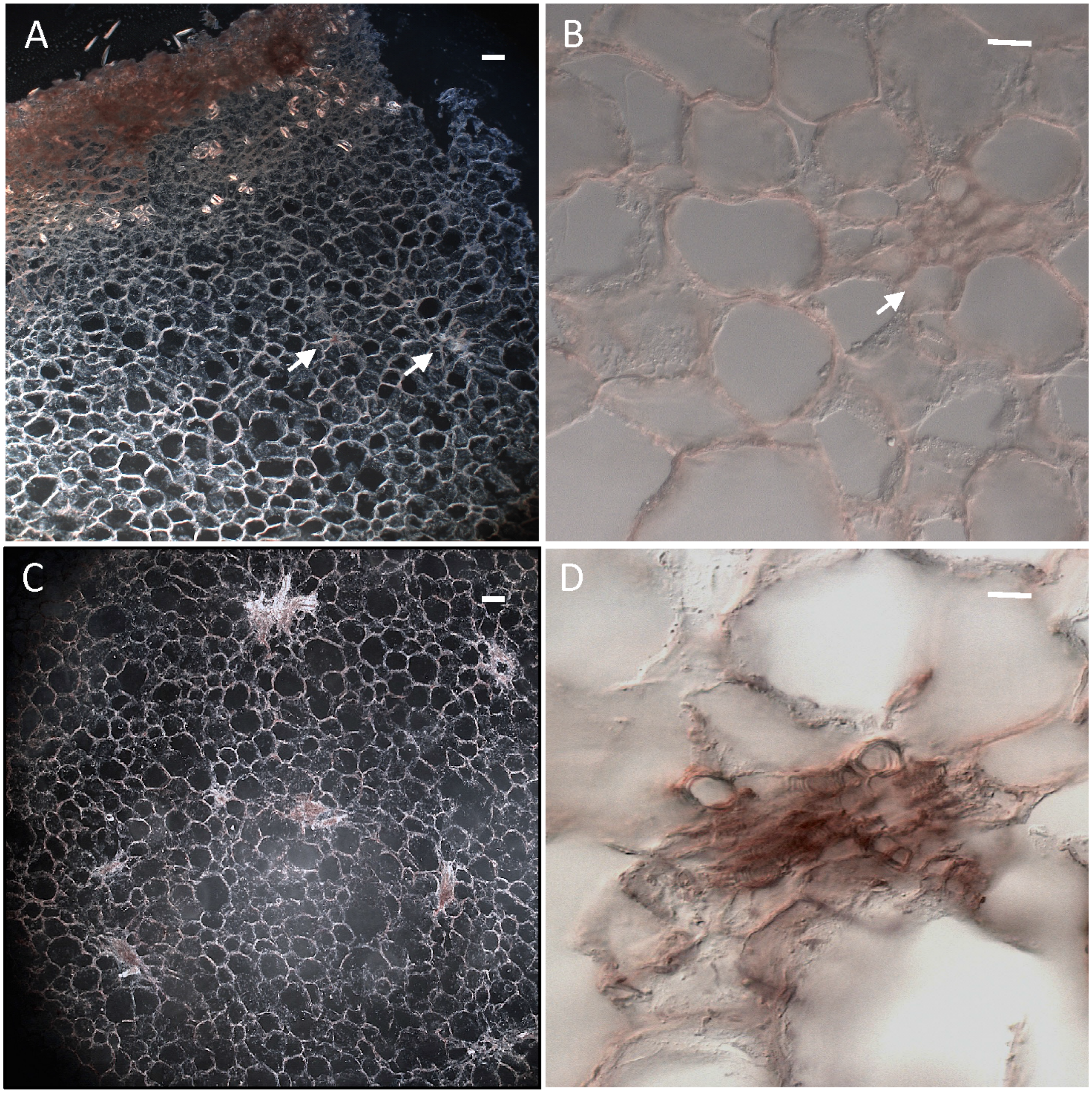

Supplementary Figure 2. Localization of *CTG134* expression in peach mesocarp by *in-situ* hybridization (control panels). (C, D) In peach mesocarp at S4, *CTG134* expression was mainly associated with vascular bundles (antisense probe), while it was not detected in control sections (A and B, sense probe). Arrows indicate where *CTG134* should be). Scale bar in the panels A and C = 200 μm, in B and D = 50 μm.

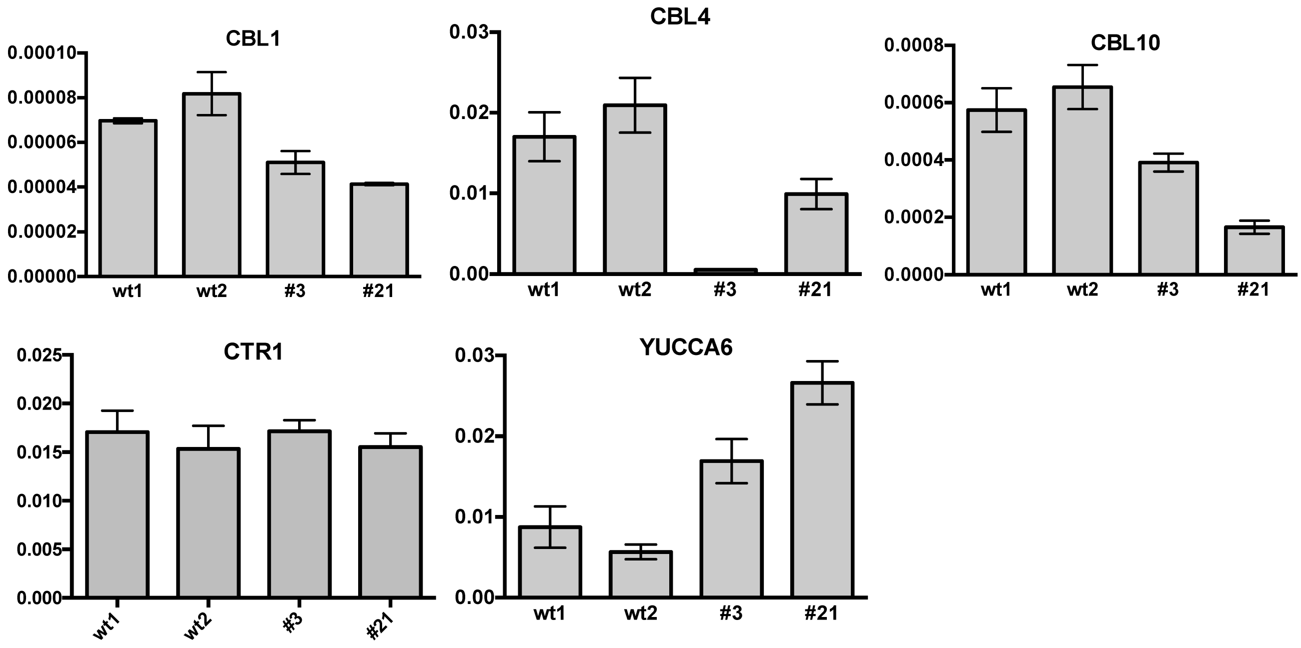

Supplementary Figure 3. Relative expression profiles of selected genes in roots of *Arabidopsis* seedlings grown on agar plates for five days. wt1 and wt2 are wild type samples collected from two different plates, while #3 and #21 are the clone identifiers of the *Arabidopsis* lines overexpressing the peach *CTG134* gene. Values (means of the normalized expression) have been obtained by real-time qRT-PCR analyses. Bars are the standard deviations from the means of three replicates. CBL1, 2 and 3 are Calcineurin B-like Calcium sensors; YUC6 is one of the two forms of the enzymes involved in the two step pathway of auxin synthesis; CTR1 is a negative regulator in the ethylene signal transduction pathway, positioned immediately after the receptors.

Supplementary Table 1. Primers used in this research.

At/ppa/Prupe code	*Primer Name*	*Forward*	*Reverse*
AT1G79840	GLABRA2 qRT-PCR	GCTGAACCAGGAAGAGTACG	CAGGTGAAGTGCACCATTTC
AT2G46410	CAPRICE qRT-PCR	AGGTGAGTAGTATCGAATGG	AAGTCTCTTCGTCTGTTGGC
AT1G04610	YUCCA3 qRT-PCR	CATCGATGGTCGTTCGTAGC	CAAGAACCGGAGTTTTGCC
AT5G25620	YUCCA6 qRT-PCR	CGTACCCTCTTGGCTAAAG	GCTCGTCTTGTTTCCAACAC
AT4G37490	CYCB1;1 qRT-PCR	GTCAAGTTCTTGGTGATATAGG	CTTCTCTAAACCACAAGCAGC
AT3G20770	EIN3 qRT-PCR	CTGTGACTGGTGCTTCTG	TTCTCCGCTTGATACTTGG
AT1G66340	ETR1 qRT-PCR	GATGCTTCCTTCAGATAGTG	CATGTGAGAGAGCTACAGCCAC
AT5G57090	PIN2 qRT-PCR	ATGAGGAAGTTATGAAGACGGCG	TTGACTCCACTTGCTCCACTCG
AT1G01480	ACS2 qRT-PCR	GGTCTTAAGAAGTTTAGACAG	GAACATGATTGTTTCATTGGC
AT1G08980	AMI1 qRT-PCR	GGACTTACTCCAATGGCTCAG	CCACGGATCCAACCAGAGGC
AT1G51760	IAR3 qRT-PCR	GCTGTTACTGGTGTTGTTGG	CTGTAGCTCTTCTTCATGTTC
AT5G03730	CTR1 qRT-PCR	CTGAGTATGGCTTATGATGTG	CTGAGTATGGCTTATGATGTG
AT4G17615	CBL1 qRT-PCR	GTCAAGCAAATGTTGATCGC	CTGAGATATGGAAGAGTCAT
AT5G55990	CBL2 qRT-PCR	ATGTCGCAGTGCGTTGACGG	CTTGTTTATTAGCCCATCATC
AT5G24270	CBL4 q-RT-PCR	GAAAGAGATGGTAGTAGCG	GATATGGCAAAGTCATGTTC
AT4G33000	CBL10 qRT-PCR	GATCAAGCTCTCTCACTGTC	GTGAATCAAGCCGTCATC
AT4G36800	RUB1 qRT-PCR	CTGTTCACGGAACCCAATTC	GGAAAAAGGTCTGACCGACA
AT1G49240	ACTIN8 qRT-PCR	CTCAGGTATTGCAGACCGTATGAG	CTGGACCTGCTTCATCATACTCTG
AT4G05320	UBIQUITIN10 qRT-PCR	GGAAAAAGGTCTGACCGACA	CTGTTCACGGAACCCAATTC
ppa012311/Prupe.7G256100	CONTIG134 in situ probe	CCACAACCACTAACACCCCTTCAA	TTAGCTTTCGCATCACCATCTTCC
ppa012311/Prupe.7G256100	ctg_134_for/ctg_134_rev	CCACAACCACTAACACCCCTTCAA	TTAGCTTTCGCATCACCATCTTCC
ppa009483m/Prupe.8G137600	PpN1	CCAGGAGAATCGGTGAGCAGAAAA	TCGAGGGTGGAGGACTTGAGAATG
